# Supplementary material for: Genetic, lifestyle and environmental influences on health: a Finnish biobank recall study protocol (BioRecall)
Source: BMJ Open. 2026 Feb 17;16(2):e100092. doi: 10.1136/bmjopen-2025-100092 (PMC12918690; doi:10.1136/bmjopen-2025-100092)
Supplement: Supplementary file 1 [file bmjopen-2025-100092.reviewer_comments.pdf]

PEER REVIEW HISTORY

BMJ Open publishes all reviews undertaken for accepted manuscripts. Reviewers are asked to complete a checklist review form and are provided with free text boxes to elaborate on their assessment. These free text comments are reproduced below.

ARTICLE DETAILS

Title (Provisional)

Genetic, lifestyle and environmental influences on health: A Finnish biobank recall study protocol (BioRecall)

Authors

Sillanpää, Elina; Föhr, Tiina; Kurtti, Elisa; Aittola, Kirsikka; Makela, Johanna; Southerington, Tom; Lakka, Timo A; Jokela, Tiina; Ahtiainen, Maarit; Laakkonen, Eija; Rantakokko, Merja; Ravi, Suvi

---

VERSION 1 - REVIEW

**Reviewer**	**1**
**Name**	**Lee, David**
**Affiliation**	**New York University Grossman School of Medicine**
**Date**	**11-Mar-2025**
**COI**	**None**

BMJ Open Review

Study seeks to describe the protocol for a Finnish study that will collect data on lifestyle and environmental exposures to understand influences on chronic disease. Study will invite all previously genotyped donors of a biobank that have diagnosed type 2 diabetes.

Strengths include prior banked samples leading to a low-cost, scalable approach, and extensive analysis of lifestyle and environmental exposures. Limitations include the author state no collection of wearable data, but that would also include that most of the assessments are self-report which is subject to limitations.

The introduction is well written and provides a good understanding for the need of this study.

Regarding eligibility: only having one diabetes code could be problematic as there may be false positives. Is there any data to suggest in the Finnish population that one diagnosis code is sufficient? Otherwise, a sensitivity analysis might be needed to study at least two or more diagnosis codes.

Given the number of questionnaires involved, what is the expected response rate? Low rates would lead to non-response bias and also potentially a sample size that is too small. Authors state: "Since the primary focus of the project is on piloting the operational model, no formal sample size calculation has been performed, and no detailed statistical analysis plan has been developed." That sounds more like a feasibility study rather than a protocol. Otherwise, if the protocol will need to be significantly adapted then it might not require pre-publishing as this is not a clinical trial.

The methods section reads as a list of different instruments that will be performed, but there is no section on how all this data will be analyzed. How will participants be incentivized? There is a lot of key details lacking. If this is all to be figured out by a feasibility study, then do the feasibility study, then create a protocol which is informed by that feasibility study.

Self-report is a key limitation not well addressed by the manuscript.

Overall, it is hard for me to understand why there is a need to pre-publish a protocol for a pilot. The manuscript also lacks a lot of details that will likely be investigated through this pilot.

Reviewer 2

Name Dzakpasu, Francis Q S

Affiliation Swinburne University of Technology - Hawthorn Campus, Department of Biomedical, Exercise & Health Sciences

Date 13-Nov-2025

COI None

This is a well-written and innovative BioRecall study concept protocol. The document is transparent on the necessary components of the proposed BioRecall concept. However, the authors may consider these few minor comments or suggestions, if they find them helpful.

1. This statement in the abstract: "Biobanks typically lack sufficient lifestyle and environmental data, which limits their use." Is this referring to biobanks in general or just the current biobank in Finland? When almost all biobanks in the near future have comprehensive datasets, including lifestyle and environmental data, this statement may become redundant in the protocol, which may have already been published.

2. Figure 1: it may be helpful to some readers if the solid and broken arrows are defined.

3. This sentence may need clarification (Page 7, Lines 14 – 16): "Lifestyle and environmental data will be collected by an electronic survey and from national open-source registers through a postal code linkage." Are the lifestyle and environmental data going to be

collected using an electronic survey and open-source registers, respectively? The protocol is clear about using electronic surveys, but a little unclear about open-source registers.

4. The latter part of this sentence seems incomplete and unclear (Page 8, Lines 48 – 52): "We plan to send two electronic reminders within a month and, if the response rate is low, paper questionnaires and prepaid return envelopes."

5. Page 10: From the items to assess muscle-strengthening activities, would it be possible to estimate the average amount of time spent on muscle-strengthening activities per day or week?

6. While the authors stated the data will be used in several MSc (Master's?) theses, which will be published in the University's digital repository (Page 15), there is no detailed description on dissemination under the "Ethics and dissemination" (Page 16) subheading. I suggest the authors delete the dissemination from the subheading.

VERSION 1 - AUTHOR RESPONSE

Reviewer: 1
Dr. David Lee, New York University Grossman School of Medicine, New York University School of Medicine
Comments to the Author:
BMJ Open Review

Study seeks to describe the protocol for a Finnish study that will collect data on lifestyle and environmental exposures to understand influences on chronic disease. Study will invite all previously genotyped donors of a biobank that have diagnosed type 2 diabetes.

Strengths include prior banked samples leading to a low-cost, scalable approach, and extensive analysis of lifestyle and environmental exposures. Limitations include the author state no collection of wearable data, but that would also include that most of the assessments are self-report which is subject to limitations.

The introduction is well written and provides a good understanding for the need of this study.

We deeply thank the reviewer for taking the time to review our paper and for the insightful comments that helped us to improve the content.

Regarding eligibility: only having one diabetes code could be problematic as there may be false positives. Is there any data to suggest in the Finnish population that one diagnosis code is sufficient? Otherwise, a sensitivity analysis might be needed to study at least two or more diagnosis codes.

Type 2 diagnosis codes are based on ICD-10 classification, which is used in Finnish healthcare. The included 11 ICD codes, presented in the manuscript pages 6-7, were E11, E11.0, E11.1, E11.2, E11.3, E11.4, E11.5, E11.6, E11.7, E11.8, or E11.9. These codes encompass well T2D without complications (E11.9) and with specific complications across body systems, while excluding, for example, Type 1 diabetics. In this pilot, we wanted to focus specifically on type 2

diabetics, whose condition is dependent on both genetics and lifestyle.

Given the number of questionnaires involved, what is the expected response rate? Low rates would lead to non-response bias and also potentially a sample size that is too small. Authors state: “Since the primary focus of the project is on piloting the operational model, no formal sample size calculation has been performed, and no detailed statistical analysis plan has been developed.” That sounds more like a feasibility study rather than a protocol. Otherwise, if the protocol will need to be significantly adapted then it might not require pre-publishing as this is not a clinical trial.

The current manuscript describes the study protocol. As pointed out, the study allows the feasibility of this type of data collection to be analysed. However, in our opinion, it does not lessen the importance of pre-publishing the study protocol. Publishing a research protocol article ensures transparency and reproducibility, allowing peers to critically assess the methodology before results are reported. This practice reduces publication bias, strengthens scientific integrity, and promotes ethical standards. Moreover, it increases the visibility of the research early on, fostering collaboration and attracting potential funding opportunities. These benefits apply to all kinds of studies, not only to clinical trials.
Based on our previous experience in recruiting participants within the same study area, and in other population-based studies, we expected to have a response rate around 30%, which would make a little bit over 474 participants (out of 1,580 participants eligible for the study). This population would provide an adequate sample to conduct statistical analysis in terms of several health-related outcomes. We plan to perform power calculations for different outcome variables whenever we publish data from the study. This information has now been complemented on page 15.

The methods section reads as a list of different instruments that will be performed, but there is no section on how all this data will be analyzed. How will participants be incentivized? There is a lot of key details lacking. If this is all to be figured out by a feasibility study, then do the feasibility study, then create a protocol which is informed by that feasibility study.

We apologize for being unclear. We plan to motivate participants through personalized feedback. This has been described under the chapter “Automatic feedback” which may be misleading. This information is placed after the information on measurement instruments, because personalized feedback is produced to the participants based on instruments used in the study. We have now rephrased the title and slightly modified the text.
Page 14:

”Participant incentivization and automatic feedback

We have developed an automatic feedback function for the REDCap software that provides participants with feedback on their personal lifestyle habits in relation to national lifestyle recommendations for health promotion and work stress. This was done to make the questionnaire more engaging for participants and to motivate invited individuals to complete the questionnaire, thus potentially increasing the response rate.” …

Self-report is a key limitation not well addressed by the manuscript.

We acknowledge the limitations of self-reports; however, collecting exclusively objective data is not always feasible. To minimize bias resulting from self-reports, we have used validated questionnaires whenever possible. Our plans include data collections using wearables, but this integration is dependent on the success of external research funding applications currently under evaluation. Health outcomes in this data originate from the Finnish health registers, which are of high quality. We have now highlighted this limitation in the discussion.

Page 17.

"In addition, self-reported lifestyle data, particularly physical activity, is susceptible to multiple sources of bias. We attempted to minimize these biases by using validated questionnaires whenever possible; however, the potential for error cannot be entirely eliminated."

Overall, it is hard for me to understand why there is a need to pre-publish a protocol for a pilot. The manuscript also lacks a lot of details that will likely be investigated through this pilot.

While this is a pilot for a larger biobank study, there will be several publications and master's thesis using this BioRecall data in the future. The purpose of this protocol paper is to provide detailed information about the full study project, to avoid unnecessary explanation in later publications. Thus, we consider that publishing the protocol paper is needed in a research project like this.

Reviewer: 2
Dr. Francis Q S Dzakpasu, Swinburne University of Technology - Hawthorn Campus
Comments to the Author:
This is a well-written and innovative BioRecall study concept protocol. The document is transparent on the necessary components of the proposed BioRecall concept. However, the authors may consider these few minor comments or suggestions, if they find them helpful.

1. This statement in the abstract: "Biobanks typically lack sufficient lifestyle and environmental data, which limits their use." Is this referring to biobanks in general or just the current biobank in Finland? When almost all biobanks in the near future have comprehensive datasets, including lifestyle and environmental data, this statement may become redundant in the protocol, which may have already been published.

Thank you for this comment. Some large biobanks, such as UKBB, include a wide variety of phenotypes. Their data collection is based on recruiting volunteers and thus is not representative of the whole population. However, most of the other biobank studies use a different approach, collecting samples via the healthcare system or via other approaches that do not entail an extensive in-person examination at recruitment. For this reason, it is often difficult to obtain extensive behavioural and lifestyle information from biobank participants and recontacting after recruitment is required. This problem is related to the Finnish biobanks in general, because biobank samples are collected during healthcare visits. For example, our national effort Finngen (https://www.finngen.fi/en), which combines data from all Finnish biobanks and 520,000 citizens, lacks lifestyle and environmental data, although for genotyping and registers, it is among the best in the world. We have modified the sentence to clarify that this is our national problem.

Abstract:

“Finnish biobanks typically lack sufficient lifestyle and environmental data, which limits their use.”

2. Figure 1: it may be helpful to some readers if the solid and broken arrows are defined.
Thank you for this good advice. We have now added definitions. Please see page 6.

“The solid line represents the sequential progression of the process from one stage to the next. The dashed line represents the availability of biobank registry data for researchers and the subsequent incorporation of collected data into the biobank registry.”

3. This sentence may need clarification (Page 7, Lines 14 – 16): “Lifestyle and environmental data will be collected by an electronic survey and from national open-source registers through a postal code linkage.” Are the lifestyle and environmental data going to be collected using an electronic survey and open-source registers, respectively? The protocol is clear about using electronic surveys, but a little unclear about open-source registers.

Thank you for the possibility to clarify this. Lifestyle data is collected using the electronic survey. Environmental data are collected using both the questionnaire and by complementing the dataset with open-source data, as described on page 13 under chapter “Residential environment”. For that purpose, we are collecting the postal numbers in the questionnaire. This has been clarified as follows:

Page 7.

“Lifestyle data will be collected by an electronic survey. The survey also includes a few questions on the perceived environment. Most of the environmental data will be collected from national open-source registers through a postal code linkage.”

4. The latter part of this sentence seems incomplete and unclear (Page 8, Lines 48 – 52): “We plan to send two electronic reminders within a month and, if the response rate is low, paper questionnaires and prepaid return envelopes.”

Thank you for the comment, the sentence is indeed unclear. We have revised our protocol and corrected the text as follows (page 7):

“One month after the research invitation, a reminder will be sent to those who have not responded at all or who have responded only partially to the electronic survey.”

5. Page 10: From the items to assess muscle-strengthening activities, would it be possible to estimate the average amount of time spent on muscle-strengthening activities per day or week?

In many studies, adherence to physical activity guidelines is examined solely in terms of aerobic exercise; our research enables the assessment of guideline adherence with respect to muscle-strengthening activities as well. Therefore, we ask how many days a week participants engage in muscle-strengthening activities. Furthermore, adherence to the strength training recommendations set by the American College of Sports Medicine (ACSM) will also be asked. This is because days per week are not informative in evaluating if the strength training is intensive enough to increase muscle strength and mass. Unfortunately, we do not have

information on the exact time (hours or minutes) spent on these activities per day. In our opinion, the time spent in muscle-strengthening activities is not as meaningful as the frequency and intensity.

6. While the authors stated the data will be used in several MSc (Master's?) theses, which will be published in the University's digital repository (Page 15), there is no detailed description on dissemination under the "Ethics and dissemination" (Page 16) subheading. I suggest the authors delete the dissemination from the subheading.

Thank you for this comment. This chapter is needed based on the journal policy, and the editor and reviewer 1 encourage us to complement this section. This has now been done and the text in the previous chapter has been slightly revised. Please see page 15.

"The project metadata will be published on the Jyväskylä University Digital Repository 'JYX'. Data will be archived in the Central Finland Biobank, where it will be made available for researchers through the Fingenious service (https://site.fingenious.fi/en/). Data will be used in scientific articles and several Master's theses. We will follow Plan S and publish in open-access journals and/or parallel-publish in the university repository 'JYX', where theses will also be published."

Reviewer: 1
If you have selected 'Yes' above, please provide details of any competing interests.: Not applicable

Reviewer: 2
If you have selected 'Yes' above, please provide details of any competing interests.: 'Not applicable'

VERSION 2 - REVIEW

**Reviewer**	**2**
**Name**	**Dzakpasu, Francis Q S**
**Affiliation**	**Swinburne University of Technology - Hawthorn Campus, Department of Biomedical, Exercise & Health Sciences**
**Date**	**03-Feb-2026**
**COI**	

The authors have done well to address my concerns. No further comments.

Thank you.
